# In Silico and In Vitro Exploration of Poziotinib and Olmutinib Synergy in Lung Cancer: Role of hsa-miR-7-5p in Regulating Apoptotic Pathway Marker Genes

**DOI:** 10.3390/medicina59111923

**Published:** 2023-10-30

**Authors:** Salman Alamery, Anfal AlAjmi, Tanveer A. Wani, Seema Zargar

**Affiliations:** 1 Department of Biochemistry, College of Science, King Saud University, Riyadh 11451, Saudi Arabia; salamery@ksu.edu.sa (S.A.); 439203099@student.ksu.edu.sa (A.A.); 2 Department of Pharmaceutical Chemistry, College of Pharmacy, King Saud University, Riyadh 11451, Saudi Arabia; twani@ksu.edu.sa

**Keywords:** poziotinib, olmutinib, lung cancer, tyrosine kinase inhibitor, STK-11, Bcl-2, Bax

## Abstract

*Background and objectives*: Non-small cell lung cancer (NSCLC) is often caused by EGFR mutations, leading to overactive cell growth pathways. Drug resistance is a significant challenge in lung cancer treatment, affecting therapy effectiveness and patient survival. However, combining drugs in research shows promise in addressing or delaying resistance, offering a more effective approach to cancer treatment. In this study, we investigated the potential alterations in the apoptotic pathway in A549 cells induced by a combined targeted therapy using tyrosine kinase inhibitors (TKIs) olmutinib and poziotinib, focusing on cell proliferation, differential gene expression, and in silico analysis of apoptotic markers. *Methods:* A combined targeted therapy involving olmutinib and poziotinib was investigated for its impact on the apoptotic pathway in A549 cells. Cell proliferation, quantitative differential gene expression, and in silico analysis of apoptotic markers were examined. A549 cells were treated with varying concentrations (1, 2.5, and 5 μM) of poziotinib, olmutinib, and their combination. *Results:* Treatment with poziotinib, olmutinib, and their combination significantly reduced cell proliferation, with the most pronounced effect at 2.5 μM (*p* < 0.005). A synergistic antiproliferative effect was observed with the combination of poziotinib and olmutinib (*p* < 0.0005). Quantitative differential gene expression showed synergistic action of the drug combination, impacting key apoptotic genes including STK-11, Bcl-2, Bax, and the Bax/Bcl-2 ratio. In silico analysis revealed direct interactions between EGFR and ERBB2 genes, accounting for 77.64% of their interactions, and 8% co-expression with downstream apoptotic genes. Molecular docking indicated strong binding of poziotinib and olmutinib to extrinsic and intrinsic apoptotic pathway markers, with binding energies of −9.4 kcal/mol and −8.5 kcal/mol, respectively, on interacting with STK-11. *Conclusions*: Combining poziotinib and olmutinib therapies may significantly improve drug tolerance and conquer drug resistance more effectively than using them individually in lung cancer patients, as suggested by this study’s mechanisms.

## 1. Introduction

Neoplasms of the lungs are the second most common cancer in men after prostate cancer and women after breast cancer [1,2]. One of the cancer hallmarks is the resistance towards the cell death mechanisms as well as the acquired resistance to the drugs used. Cancer cells often modulate death mechanisms by overexpressing antiapoptotic proteins to neutralize proapoptotic proteins [3]. Lung cancer is a highly prevalent cancer worldwide, accounting for 11.4%, and has a poor prognosis, with 5-year survival rates of 4% to 17% depending on the stage at the time of diagnosis [4]. Moreover, the recently projected statistics for lung cancer in the United States in 2023, as per the American Cancer Society, are approximately 238,340 fresh incidences of lung cancer, which include an estimated 127,070 fatalities [5]. Although non-invasive tests have improved the chances of identifying lung cancer, only 10–15% of newly formed neoplasms are discovered at an early clinical stage. Lung cancer is detected in 75% of patients at an advanced stage when treatment options are restricted with gradually increasing genetic and epigenetic changes [6]. Non-small cell lung cancer (NSCLC) constitutes nearly 85% of all lung cancer cases and is characterized by the presence of oncogenic driver mutations [7]. However, the development of resistance to targeted therapies in these tumors, driven by a multitude of mutations, remains a complex and poorly understood process. Among epidermal growth factor-tyrosine kinase (EGFR-Tk)-mutated lung cancers, the most common resistance-inducing mutation is T790M on exon 20.

Fortunately, there are now highly potent third-generation tyrosine kinase inhibitors (TKIs) available to effectively combat this mutation [8]. The T790M mutation, a TKI-sensitive EGFR mutation showing resistance after 9 to 13 months of TKI treatment, was found to be present in approximately 50% to 60% of resistant cases of NSCLC [9].

Different therapies target different pathways of cells to regulate cancer progression. Some agents target monoclonal antibodies and others inhibit multiple kinase receptors, such as human epidermal growth factor receptor 2 (HER2), fibroblast growth factor receptor (FGFR), anaplastic lymphoma kinase (ALK), and epidermal growth factor receptor (EGFR). The ultimate goal of the treatment is palliation, by improving the quality of patient life and prolonging patient survival [10,11,12]. STK-11, also referred to as liver kinase B1 (LKB1), is a pivotal serine–threonine protein kinase that assumes the role of a critical tumor suppressor gene. It is frequently found to harbor mutations in a diverse spectrum of human cancers [13]. This serine–threonine kinase possesses the direct ability to phosphorylate and regulate not only the adenosine monophosphate-activated protein kinase (AMPK) but also a dozen other kinases closely resembling AMPK. Functioning as a tumor suppressor gene, STK-11 exerts influence over various fundamental biological processes, including the regulation of cell migration, polarity, apoptosis, and proliferation. Moreover, it exerts a regulatory effect on apoptosis within cancer cells through the modulation of the expression and activity of key apoptotic proteins such as BCL-2, BAX, and cleaved caspase. These actions are pivotal in governing the PI3K/AKT/mTOR signaling pathway, which in turn plays a critical role in the realms of cell survival, proliferation, metabolism, and angiogenesis [14]. Third-generation TKIs have been designed to target more effectively the T790M mutations [15]. STK-11/LKB1 is a multifaceted protein with a critical role in cell regulation, energy sensing, and tumor suppression. Its significance in cancer, particularly lung cancer, underscores its potential as a therapeutic target and a biomarker for tailored cancer treatment strategies. Drug resistance has been linked to mutations in the STK-11 gene in a number of cancer types, including non-small cell lung cancer (NSCLC) [16]. Vemurafenib, the first RAF inhibitor approved for clinical use, effectively inhibits the BRAF-V600E mutant [17]. Initially, vemurafenib exhibited remarkable effects, leading to the visible regression of melanoma in many patients within weeks. However, the resurgence of melanoma, driven by the proliferation of residual cells resistant to vemurafenib, curtailed this remission, with melanoma returning within weeks to months in affected patients. Similar trends were observed with dabrafenib (an RAF inhibitor) and trametinib (an MEK1 and MEK2 inhibitor) [18]. Intriguingly, when dabrafenib and trametinib were combined, there was a significant delay in the recurrence of metastatic melanoma, resulting in survival rates of 37% and 34% after 4 to 5 years, respectively, with notable complete responses reported in 19% of patients. It was found that the combination therapy of vemurafenib, dabrafenib, and trametinib significantly improved overall survival and management of metastasis in melanoma patients previously untreated with vemurafenib monotherapy [18]. Thus, combination of targeted therapies holds promise as a strategy to combat resistance to 3rd-generation TKIs in patients, and numerous clinical studies are underway to explore novel combination therapies [19]. The enhanced efficacy of this combination may be attributed to the simultaneous targeting of two kinases acting sequentially in the same signaling pathway, as well as the suppression of bypass mechanisms stemming from the activation of other RAF isoforms and RAF-MEK complexes [20]. Keeping these studies in consideration, this study aimed to investigate the efficacy of poziotinib and olmutinib alone and compare their combination intervention on attenuating the progression of NSCLC through induction of apoptosis, the main downstream event after chemotherapy. In silico analysis was used to depict the potential gene targets that enhance extrinsic and intrinsic apoptotic pathways in the lung cancer A549 cell line. The genes showing maximum interactions were selected for qPCR analysis and further analyzed for miRNA enrichments and pathways. Combining in vitro and in silico approaches may provide a comprehensive understanding of the effects and mechanisms of olmutinib and poziotinib, as well as their potential to overcome drug resistance in lung cancer. This combined research effort can guide the development of more effective and personalized treatment strategies for patients, ultimately improving their outcomes and quality of life. The pursuit of new combination therapies to combat tyrosine kinase inhibitor (TKI) resistance is a crucial step in revolutionizing cancer treatment. These innovative approaches offer the promise of bolstering TKIs’ effectiveness, thwarting resistance, customizing treatment, extending patient lifespans, and broadening therapeutic choices. They symbolize significant progress in the ongoing fight against drug resistance and the unwavering drive to deliver more efficient, tailored, and ultimately life-saving cancer treatments. The simultaneous targeting of two kinases within the same pathway is a strategic approach to overcoming resistance in cancer therapy. By disrupting redundancy, thwarting resistance mechanisms, and achieving synergistic effects, this approach offers a more comprehensive and effective means of controlling tumor growth and improving patient outcomes. The safety profiles of poziotinib and olmutinib based on clinical data show mild gastrointestinal side effects, however, healthcare providers may have detailed information about the safety and potential side effects of these medications.

## 2. Materials and Methods

### 2.1. In Silico Analysis

The bioavailability and physicochemical properties of olmutinib and poziotinib were explored using SwissADME software (http://www.swissadme.ch/). All pharmacokinetic factors including solubility, lipophilicity, absorption, distribution, metabolism, and excretion of the drugs were evaluated. Also, passive human gastrointestinal absorption (HIA), blood–brain barrier (BBB) permeation, and substrate or non-substrate of the permeability glycoprotein (Pgp) were detected by the BOILED-Egg model [21,22,23]. The GeneMANIA program (https://genemania.org/)was used to examine the potential genes that show total physical interaction with epidermal growth factor receptor (EGFR) and tyrosine kinase receptor (ERBB2) to impact the anticancerous activity and the results obtained were further refined into extrinsic and intrinsic apoptotic pathway genes [24,25]. Significantly interacting genes were evaluated for pathway and miRNA enrichment through MIENTURNET to create miRNA networks through MiRTarBase (https://mirtarbase.cuhk.edu.cn). Functional enrichment pathway analysis was conducted using the MIENTURNET web tool, which searched the WikiPathways and Disease Ontology databases. A significance cutoff of 0.05 was applied, and the Benjamini–Hochberg method was employed to adjust *p*-values. The analysis focused on identifying functional annotations that showed significant enrichment across the entire gene list, with a particular emphasis on genes associated with both intrinsic and extrinsic apoptotic pathways in the input list [26].

### 2.2. Preparation of Drug Stocks

The standard of the two drugs was obtained from Weihua Pharma Co., Ltd., Zhejiang, China. Both olmutinib and poziotinib are poorly soluble drugs, hence, 1 mg/mL of each drug was dissolved in 100% DMSO and kept at 4 °C. Three appropriate final drug concentrations were selected for cell treatments based on previous literature as 1, 2.5, and 5 µM. The drug stock solutions were diluted to obtain final concentrations accordingly in sterilized distilled water and kept in −20 °C for further cell treatments.

### 2.3. Cell Culture

A human adenocarcinoma lung cancer cell line (A549) was maintained and grown in Dulbecco’s modified Eagle’s medium (Sigma-Aldrich®, Ronkonkoma, NY, USA) supplemented with 10% fetal bovine serum (FBS) from Gibco^®^, Waltham, MA, USA and 1% penicillin–streptomycin (Promega^®^, Madison, WI, USA). Cells were incubated in a humidified atmosphere with 5% CO_2_ at 37 °C. The A549 cells were subcultured 2/3 times a week by washing with PBS and incubated for three minutes with 0.05% Trypsin/EDTA in a CO_2_ incubator to dissociate the cell monolayer. The cells were grown to more than 80% confluence and treated with 1, 2.5, and 5 µM poziotinib drug alone and the combination with the same concentrations to assess their cytotoxic effect on cell proliferation.

### 2.4. MTT Cytotoxicity Assay

MTT assay was used to estimate the cell proliferation and a safe and effective dose of a drug treatment was determined. Cell proliferation was analyzed by recording changes in absorbance at 570 nm using a microplate-reading spectrophotometer. In brief, 200 µL of cells at a concentration of 10^3^ cells per well was seeded in a 96-well plate and incubated for 24 h to allow the cells to acclimatize and attach to the wells. Cells were treated with 1, 2.5, or 5 µM of poziotinib, olmutinib alone, and both poziotinib and olmutinib for 24 and 48 h and compared with the control treated with DMSO. After treatment, 100 µL of MTT solution (5 mg/mL in PBS) per well was added followed by shaking for 5 min at 300 rpm at 25 °C and then incubated for 2 h. After incubation, culture media were removed and 100 µL DMSO was added per well with shaking at 300 rpm at 25 °C for 30 min. The absorbance of the developed purple color was measured using an X-Mark Microplate reader (Bio-Rad, Hercules, CA, USA) at 570 nm.

### 2.5. Gene Expression Analysis

Based on the MTT results, 2.5 µM treatment was chosen for qPCR assay and labeled as A549 (control), P (poziotinib), O (olmutinib), and P + O (combination). The flasks at 80% confluence were treated with 2.5 µM final concentrations of poziotinib, olmutinib, and the combination of poziotinib with olmutinib. The cells were incubated for 48 h before starting RNA extraction using the RNeasy^®^ Plus Mini Kit (QIAGEN, Germantown, MD, USA) following kit instructions. The purity and concentration of the extracted mRNA were quantified from OD ratio (A260/280 nm), using a NanoDrop ND-1000 spectrophotometer (Thermo Fisher Scientific, Epsom, UK). The total RNA was reverse transcribed using the SuperScript^®^ III First-Strand Synthesis System for RT-PCR Kit (Thermo Fisher Scientific, Waltham, MA, USA) according to the manufacturer’s protocol. The prepared cDNA was stored at −20 °C until further analysis. The mRNA expression levels of STK-11, Bcl, and Bax were measured. Briefly, qPCR reactions were prepared in a Micro Amp™ Optical 8-Tube Strip 0.2 mL from Applied Biosystems^®^ (Life Technologies, Carlsbad, CA, USA). The sequences of the primers of STK-11, Bcl, and Bax (target genes) and GAPDH primer (reference/internal control gene) are listed in Table 1. The qPCR reaction was performed using SYBR Green master mix on the SLAN^®^ Real-Time PCR System procured from Shanghai Hongshi Medical Technology, Shanghai, China. The initial concentration of RNA used was 190 ng, and each primer worked best at a 10 µL/µM concentration.

The qPCR thermocycler conditions were 15 min at 95 °C (hold stage), 15 s at 95 °C, then 30 s at 60 °C, and 30 s at 72 °C (PCR stages), followed by the post-reading stage, 5 min at 72 °C, and 5 min at 95 °C (melting curve stages). The relative gene expression values of mRNA transcripts were calculated after normalizing to the values of the GAPDH housekeeping gene and relative control samples. The data were analyzed using the comparative threshold cycle method (2^−ΔΔCT^) [27].

### 2.6. Molecular Docking Analysis

Poziotinib and olmutinib were investigated for their interactions with the STK-11, Bcl-2, and Bax genes using molecular docking analysis. The chemical structures of poziotinib and olmutinib were retrieved in SDF format from PubChem with respective CIDs of 25127713 and 54758501. These structures were subsequently converted to PDB format using Discovery Studio. The three-dimensional crystal structures of the STK-11, Bcl-2, and Bax proteins were downloaded in PDB format from the Protein Data Bank, with the PDB IDs being LKB1, 6qgh, and 5w62, respectively.

The molecular docking procedures were conducted using Autodock Vina [28] in conjunction with MGL Tools-1.5.6 (Autodock-4) [29]. Docking files were prepared using Autodock-4, while Discovery Studio served as a visualization tool. The default docking parameters were employed, with a grid box dimension of 20 Å × 20 Å × 20 Å, and the exhaustiveness was set to 8 to ensure comprehensive exploration of binding sites. All configurations resulting from the docking process underwent physical validation to confirm the accurate placement of the ligand within the binding site. The binding modes were subsequently clustered based on root mean square deviation, and comparisons of docking results were made by evaluating the binding energies of different conformations.

### 2.7. Statistical Analysis

The statistical software GraphPad Prism V.8.0, IBM SPSS, and Microsoft Excel^®^ were used to perform statistical analysis. The relative gene expression was calculated using the 2^−∆∆Ct^ method. One-way ANOVA with Tukey’s multiple comparison *t*-test was used to analyze the associations between the different groups. All *p*-values reported in the study were calculated as two-tailed, and a *p*-value of less than 0.05 was deemed statistically significant.

## 3. Results

### 3.1. In Silico Analysis Revealed Passive Human Gastrointestinal Absorption of Both Drugs and Downstream Action on the Apoptotic Pathway

The physicochemical and pharmacokinetic properties of poziotinib and olmutinib are listed in Table 2. Poziotinib has a molecular weight of approximately 486.59 g/mol and is notably well-absorbed via oral administration, featuring a more extended half-life that generally spans the range of 15–19 h. Metabolism of poziotinib predominantly occurs in the liver, where cytochrome P450 (CYP) enzymes play a vital role, and its elimination process does not involve the P-glycoprotein (Pgp) transporter and hence it is eliminated slowly in feces and urine. Conversely, olmutinib, with a molecular weight of about 491.34 g/mol, similarly exhibits favorable oral absorption and a half-life typically ranging from 13 to 17 h. Its primary metabolic route is the liver, principally mediated by CYP3A4 enzymes, and elimination occurs through the Pgp transporter via feces and urine. It is important to bear in mind that the pharmacokinetic profiles of these drugs can exhibit interindividual variability, influenced by factors such as individual metabolic rates, concurrent medication use, and overall health status (Table 2).

**Figure 1:**
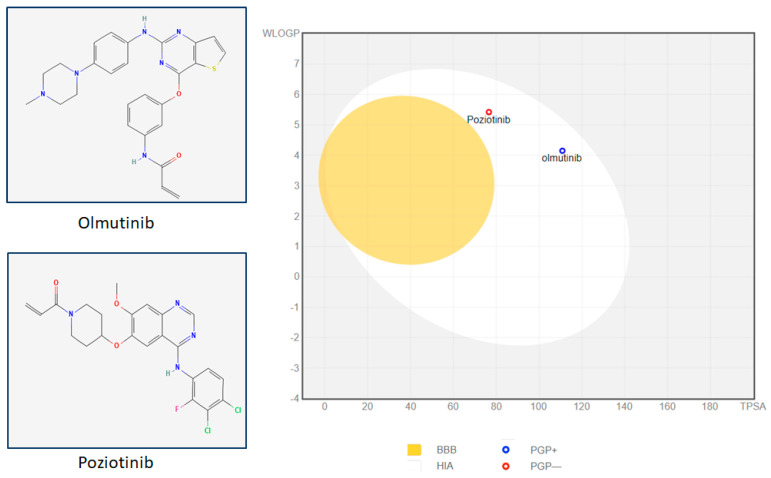
Poziotinib and olmutinib structures were adapted from PubChem and SMILES were used for BOILED-Egg demonstration in Swiss ADME’s prediction tool. Poziotinib and olmutinib are absorbed passively by gastrointestinal absorption. Both TKIs do not cross the blood–brain barrier. The olmutinib in egg white (blue dot), demonstrates it as Pgp substrate and hence could be pumped out in secretions or feces while poziotinib may retain its concentrations for longer as it is a Pgp non-substrate.

Passive human gastrointestinal absorption was observed for both olmutinib and poziotinib and both TKIs did not cross the blood–brain barrier. Additionally, the results from the BOILED-Egg graph between total polar surface area and WLogP depicted poziotinib as a Pgp non-substrate and olmutinib as a P-glycoprotein substrate. Pgp substrates are eliminated and washed out by the human digestive tract more quickly than Pgp non-substrates. Both drugs did not cross the blood–brain barrier (Figure 1).

**Figure 2:**
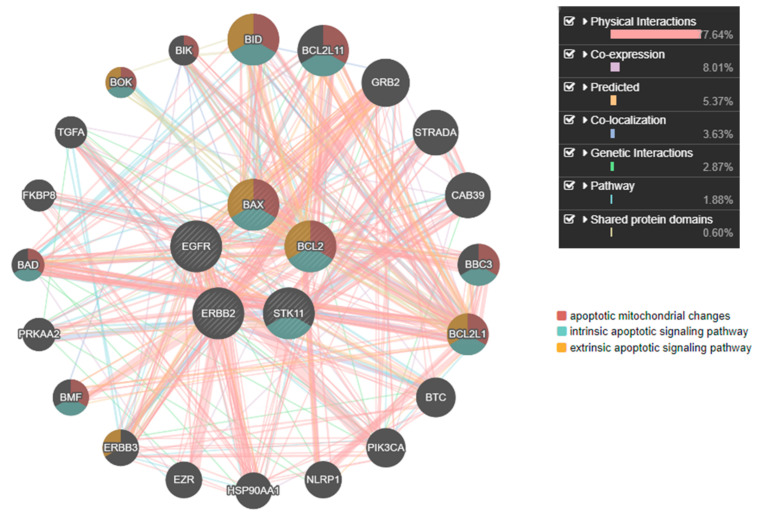
A network of epidermal growth factor receptor (Egfr) and tyrosine kinase receptor (Erbb2) interactions. EGFR and ERBB2 show maximum physical interactions (77.6%) and co-expression (8%) with bcl-2, bax, and stk-11 proteins with implicated pathways as apoptotic mitochondrial changes, intrinsic and extrinsic pathways.

GeneMANIA Cytoscape plug-in analysis provided us with protein–protein interaction networks. In this analysis, EGFR and ERBB2 showed 87.64% direct physical interactions with downstream apoptotic markers Bax, Bcl-2, and STK-11. All three downstream genes were significantly involved in activating apoptotic mitochondrial changes, intrinsic apoptotic signaling pathways, and extrinsic apoptotic signaling pathways (Figure 2).

### 3.2. Synergistic Effect of Poziotinib with Olmutinib on Cell Proliferation Activity

**Figure 3:**
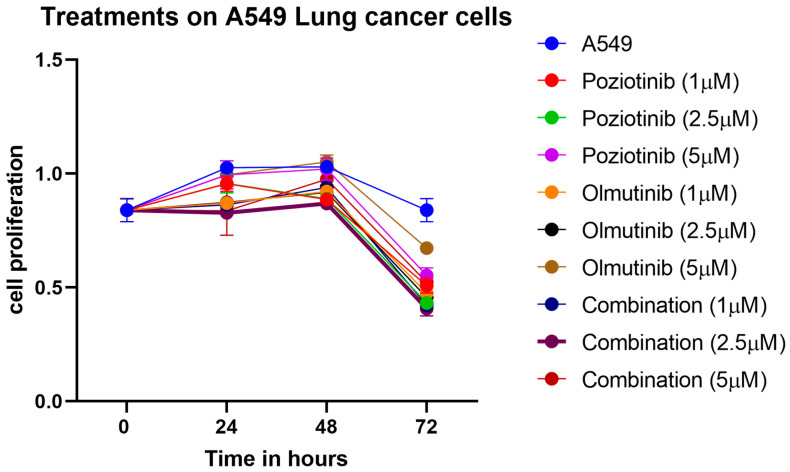
The Cell proliferation assay data in A549. Treatment of cells at concentrations of 1, 2.5, and 5 μM of poziotinib, olmutinib, and their Combination for 24 h, 48 h, and 72 h. Absorbance results represented as mean and standard error measured at 405 nm are expressed in the graph. The combination of poziotinib and olmutinib at 48 h is seen to have a maximum antiproliferative effect on A549 cells compared to all other treatments. This indicates that there was a significant reduction in cell proliferation potential with the combination (*p* < 0.05).

Reduced proliferation and cell viability were seen with the treatment of poziotinib and olmutinib on A549 cells as compared to the untreated cells. A more potent decrease was at the concentration of 2.5 µM when compared to 1 and 5 µM. The decreased proliferation was enhanced in cells undergoing combined therapy of poziotinib and olmutinib compared to cells treated with each drug alone. Results indicated that poziotinib, olmutinib, and the synergism between the two of them may have induced A549 cell apoptosis because of decreased proliferation at each concentration, maximally seen at the concentration of 2.5 μM (Figure 3).

We attempted to unveil the underlying mechanisms at the defined concentration treatments and 48 h based on apoptotic activity. GeneMANIA interaction analysis results showed maximum interactions of EGFR and tyrosine kinase with Bax, Bcl-2, and STK-11 genes (Figure 2). Hence, these genes were checked for gene expression with and without treatments.

### 3.3. Gene Expression in Response to Combined Therapy and Olmutinib and Poziotinib Alone

**Figure 4:**
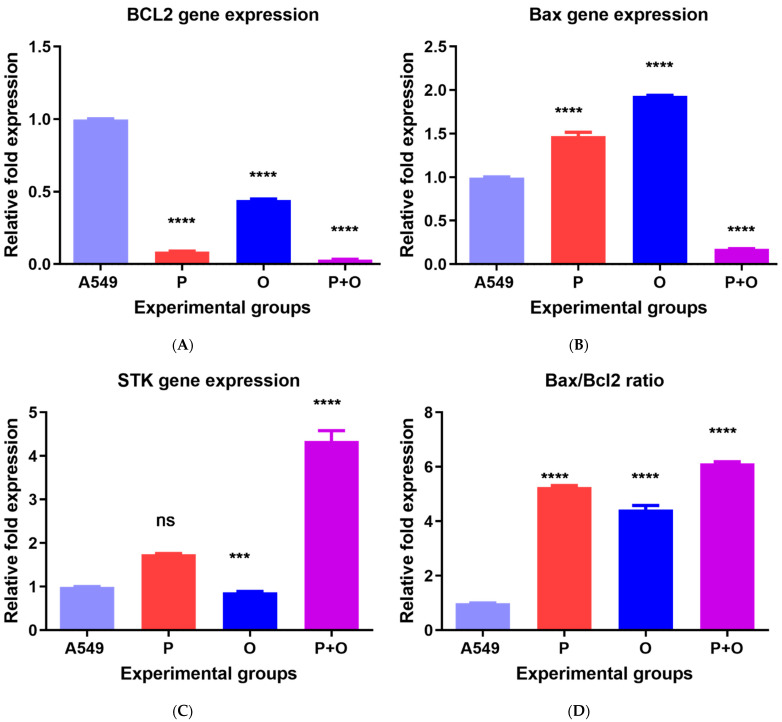
Relative gene expression levels of apoptotic markers. (**A**) Bcl; (**B**) Bax; (**C**) stk-11; and (**D**) bax/bcl-2. The expression levels of apoptotic gene markers were assessed using qPCR in cells treated with poziotinib and olmutinib alone and in combination. All assessed genes showed synergism in drug combination and better potential. Values are represented as mean ± SD of three replicates *** *p* < 0.005 and **** *p* < 0.0005, ns: not significant.

The mRNA expression level of Bax, Bcl-2, and STK-11 was measured by real-time PCR and normalized with the GAPDH expression with treatments and compared to the control cells treated with an equal concentration of DMSO used in drug solubility. The level of gene expression of all studied genes showed a variation in gene expression in response to treatments when compared to control. Poziotinib, olmutinib, and combined therapy decreased the expression of the Bcl-2 antiapoptotic gene (Figure 4A). Similarly, treatment with poziotinib and combined therapy decreased the Bax proapoptotic mRNA expression (0.42- and 0.17-fold change), while olmutinib increased the Bax expression level (1.94-fold change) (Figure 4B). The STK-11 expression also increased in response to poziotinib alone and combination therapy (1.73- and 4.14-fold change) while olmutinib alone showed a slight decrease in the expression of STK-11 (0.85-fold change) in comparison to the control cells (Figure 4C). The Bax/Bcl-2 gene expression ratio increased under the effect of poziotinib alone (5.25-fold change) and was highest after treating the cells with the combined therapy of poziotinib and olmutinib (6.06-fold change), as compared to the control cells and to the cells treated with olmutinib alone (Figure 4D).

### 3.4. Apoptotic Effects of Poziotinib and Olmutinib in Lung Cancer Cells Are Mediated by Extrinsic and Intrinsic Apoptotic Pathway Genes through hsa-miR-7-5p

**Figure 5:**
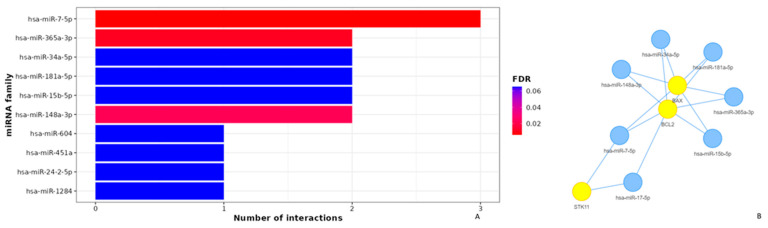
Target interaction network of miRNAs targeted by Bcl-2, bax, and stk-11 genes using MIENTURNET. (**A**) miRNA and gene Networks where each bar represents the degree of involvement of three genes in chemotherapeutic mechanisms. Three interactions were observed for miRNA-5-7p. (**B**) Significant network layout of involvement of miRNA-5-7p. The size of the nodes corresponds to the gene ratio, yellow nodes indicate genes and blue-colored nodes indicate miRNAs. Hsa-miRNA-5-7p showed significant interactions with all three apoptotic markers.

Ten miRNAs were found to regulate bcl-2, bax, and stk-11 target genes by poziotinib and olmutinib. The degree of miRNA interactions was obtained with a maximum degree in hsa-miR-7-5p for all three gene targets (Figure 5A). Hence, both extrinsic and intrinsic apoptotic pathway genes as mentioned were differentially expressed by their interaction of hsa-miR-7-5p with a false discovery rate of approximately less than 0.005. The interaction of hsa-miR-7-5p with all three genes was shown as an interaction network with yellow nodes and hsa-miR-7-5p as blue nodes (Figure 5B).

**Figure 6:**
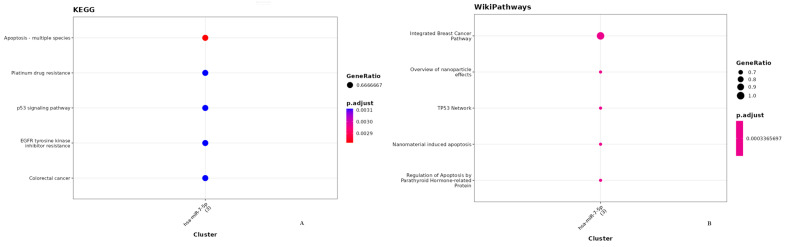
Reactome and WikiPathways Enrichment Analysis of the pathways targeted by miRNA-5-7p. (**A**) The key pathways involved in the therapeutic synergistic action of poziotinib and olmutinib are apoptosis, platinum drug resistance pathway, p53-dependent apoptotic pathway, EGFR tyrosine kinase resistance pathway, and colorectal cancer pathway. (**B**) The mechanisms of regulation were related to the integrated breast cancer pathway at a *p*-value of 0.0003. The enrichment of the network’s miRNAs is shown as dot plots with the annotation on the Y-axis and the miRNAs on the X-axis, the number (3) in the bracket after the miRNA name is the number of identified targets. The size of each dot corresponds to the gene ratio with FDR.

It was observed that apoptosis plays a significant critical role in the therapeutics of both TKIs with a *p*-value less than 0.002 by the hsa-miR-7-5p regulation reactome (Figure 6A). It was also depicted that this type of regulation was related to an integrated breast cancer pathway by WikiPathways (Figure 6B).

### 3.5. Molecular Docking Analysis

**Figure 7:**
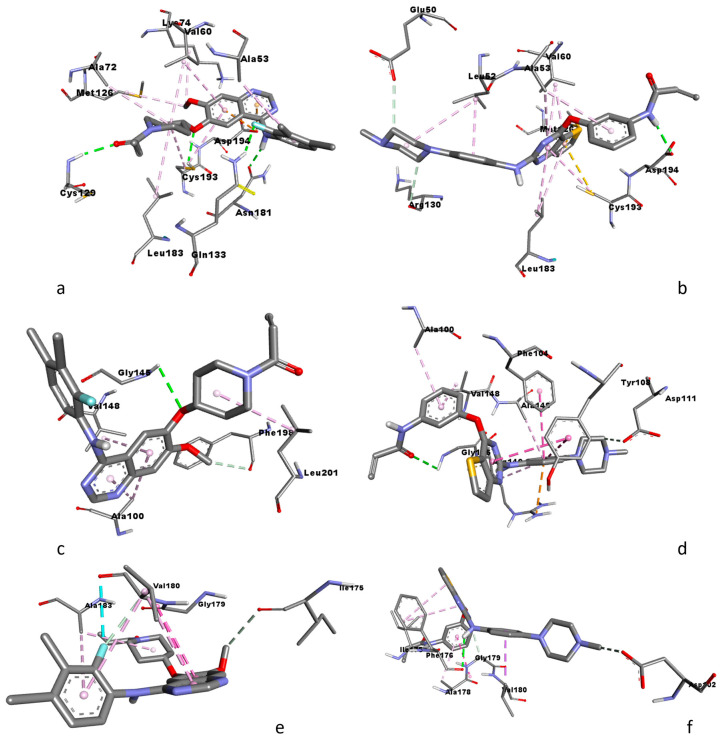
Molecular docking conformations of (**a**) poziotinib and STK-11; (**b**) olmutinib and STK-11; (**c**) poziotinib and Bcl-2; (**d**) olmutinib Bcl-2; (**e**) poziotinib and Bax; (**f**) olmutinib and Bax.

Molecular docking was conducted to assess the interactions between tyrosine kinase inhibitors, poziotinib and olmutinib, and the active sites of the crystal structures of STK-11-, Bcl-2-, and Bax-binding proteins, which corresponded to LKB1, 6qgh, and 5w62, respectively. The most suitable conformations of the interactions are given in Figure 7. The computed binding energies indicated that the interaction of poziotinib with STK-11 yielded an energy of −9.4 kcal/mol, while its interaction with olmutinib resulted in an energy of −8.5 kcal/mol. In contrast, the interaction of Bcl-2 with poziotinib produced an energy of −8.1 kcal/mol, and its interaction with olmutinib showed an energy of −8.7 kcal/mol. For Bax, the binding energies were −5.6 kcal/mol for poziotinib and −6.2 kcal/mol for olmutinib.

Based on the binding energies, poziotinib exhibited a more stable complex with STK-11 compared to olmutinib. Conversely, olmutinib formed more stable complexes with Bcl-2 and Bax when compared to poziotinib.

Analyzing the hydrogen bond interactions, poziotinib formed four hydrogen bonds with STK-11 at specific residues, namely CYS129, GLN133, CYS193, and ASN181, with bond lengths of 2.23704, 2.53819, 2.41956, and 2.16198, respectively. In the case of olmutinib, it formed three hydrogen bonds with STK-11 at ASP194, GLU50, and ARG130, with respective bond lengths of 2.16801, 3.50033, and 3.63356. In terms of interactions with Bcl-2, poziotinib established two hydrogen bonds with the residues GLY145 and PHE194, featuring bond lengths of 2.89945 and 3.60901, respectively. Olmutinib also formed two hydrogen bonds with Bcl-2, involving the residues GLY145 and ASP111, with bond lengths of 2.88527 and 3.50715. Examining the interactions with Bax, poziotinib formed two hydrogen bonds with VAL180 and ILE175, displaying bond lengths of 3.08055 and 3.47278. Olmutinib, on the other hand, formed three hydrogen bonds with Bax, engaging the residues PHE176, GLY179, and ASP102, with bond lengths of 2.48767, 3.45186, and 3.33357, respectively.

## 4. Discussion

EGFR exon 20 alterations in non-small cell lung cancer (NSCLC) present a formidable treatment challenge due to their limited therapeutic options and resistance to several tyrosine kinase inhibitors (TKIs). To address this issue, numerous research studies have explored innovative combination therapies. In one notable clinical trial, a combination of erlotinib and cetuximab, both of which are EGFR inhibitors, exhibited significant potential in enhancing EGFR targeting. Notably, this combination therapy yielded a remarkable outcome, with a patient carrying an EGFR exon 20 insertion alteration still demonstrating a partial response even after 42 months of treatment. This breakthrough offers hope for improving the prognosis of NSCLC patients with EGFR exon 20 alterations [30]. In a separate research investigation, it was documented that cetuximab might have the capacity to enhance the effectiveness of afatinib or osimertinib in patients with non-small cell lung cancer (NSCLC) harboring EGFR exon 20 insertion mutations. This combination treatment demonstrated a high level of tolerance and efficacy in in vivo studies [31]. In this study, we investigated the role of tyrosine kinase inhibitor drugs poziotinib and olmutinib alone, and the synergy of poziotinib with olmutinib, in non-small cell lung cancer cells (A549 cell line). Receptor tyrosine kinases (RTKs) are the main downstream targets of cell processes including extracellular environmental changes, cell proliferation, differentiation, migration during metastatic process or wound repair, apoptosis, and angiogenesis. After the binding of growth factors (GFs) to the EGFR, the TK activates the above-mentioned downstream signals that transmit activation signals to the nucleus and elicit the responses [32]. As expected with TKIs, downregulation of proliferation was observed in the A549 cell line treated with poziotinib and olmutinib alone and an enhanced decrease was seen in combination therapy mainly at the concentration 2.5 µM. Poziotinib and olmutinib are ATP analogs that function by inhibiting EGFR signaling. They achieve this by competing with and binding to the ATP-binding pockets located within the intracellular catalytic kinase domain of the receptor tyrosine kinases (RTKs). This binding action prevents trans-phosphorylation and effectively blocks the activation of downstream signaling pathways including proliferation [33]. Previous studies reported that poziotinib (HM781-36B) is an irreversible pan-HER TKI, which targets EGFR, HER2, and ErbB4 [34]. In this study, the synergistic effect of poziotinib and olmutinib was observed in combination therapy. Consistent with these reports, a study reported that HER2-amplified gastric cancer cells and breast cancer cells have shown responsiveness to poziotinib, with an IC50 ranging from 1 to 4 nanomoles (nM) [35]. Various other studies reported that when imatinib and vinorelbine were combined, the growth inhibition was significantly increased [36]. Furthermore, when the two drugs were combined, a lower vinorelbine concentration was required to achieve the same growth inhibition as compared to the two drugs used separately [36]. In silico analysis revealed that the cell proliferation decrease in A549 was because of apoptosis by both intrinsic and extrinsic genes interacting with EGFR and ERBB2. It was previously reported that anticancer drugs eradicate cancer cells either by disrupting cellular pathways vital for cell survival or by activating programmed cell death (apoptosis). Apoptosis is the final executor of many anticancer drugs. We found that apoptosis in A549 cells was induced by extrinsic or intrinsic pathways. The extrinsic pathway is mainly involved in controlling cell turnover as well as eliminating mutant cells, while the intrinsic pathway is involved in antineoplastic drug action [37]. The intrinsic pathway was induced through B-cell lymphoma protein 2 (Bcl-2)-associated X (Bax) and Bcl-2, a promoter and an inhibitor of apoptosis, respectively, and the extrinsic pathway was induced through STK-11 overexpression. Cell proliferation and the qPCR analysis showed synergism of combination therapy. The decrease was more significant in bcl-2 gene expression and the increase in Bax and Bax/Bcl-2 ratio gene expression in combination treatment. Similarly, an increase in STK-11 expression was observed by both drug treatments, and synergism was observed by combination treatment in comparison to the control cells. Both Bax and Bcl-2 as well as their ratio have been regarded as prognostic markers in various types of cancers. The equilibrium between these two proteins, frequently gauged by determining the Bax/Bcl-2 ratio, offers valuable information regarding the capacity of cancer cells for apoptosis [38,39]. The extrinsic pathway was induced through stk-11 gene expression. It is demonstrated that stk-11 physically associates with p53 and regulates a specific p53-dependent extrinsic apoptosis pathway [40]. Stk-11 protein is present in the cytoplasm as well as the nucleus and migrates to mitochondria during apoptosis [41]. The key pathways involved in the therapeutic synergistic action of poziotinib and olmutinib were apoptosis, platinum drug resistance pathway, p53-dependent apoptotic pathway, EGFR tyrosine kinase resistance pathway, and colorectal cancer pathway. Similar mechanisms of regulation were also depicted and related to the integrated breast cancer pathway at the *p*-value of 0.0003. The development of apoptosis-targeting anticancer drugs has gained much interest since cell death induced by apoptosis causes minimal inflammation. The intricate nature of the apoptosis mechanism and how tumor cells have evolved to resist cell death have directed research efforts toward developing innovative strategies aimed at triggering apoptosis in cancer cells [42]. It was elucidated by miRNA interactions that the apoptotic pathways activated by bcl-2, bax, and stk-11 were regulated by hsa-miR-7-5p miRNA. MiR-7-5p has been identified as a crucial tumor suppressor involved in the modulation of various malignant tumor characteristics, including cell proliferation, apoptosis regulation, migration, invasion, as well as responsiveness to both chemotherapy and radiotherapy. Its significance has been noted in several cancer types, including breast cancer, non-small cell lung cancer, and nasopharyngeal carcinoma [43,44,45,46,47]. MiR-7-5p has been identified as a potent inhibitor of tumor metastasis in non-small cell lung cancer. It exerts its effects by targeting NOVA2, a protein primarily associated with neuronal migration [44]. Additionally, miR-7-5p has demonstrated its ability to impede brain metastasis in breast cancer stem-like cells, achieved through the regulation of KLF4 expression [47]. TP53-regulated inhibitor of apoptosis 1 (TRIAP1) represents a recently discovered downstream gene regulated by p53, which plays a pivotal role in mediating the antiapoptotic functions of p53. When TRIAP1 is overexpressed, it can impede the activation of APAF1/apoptosome, consequently blocking apoptosis in cancer cells. This inhibition of programmed cell death contributes to the advancement of cancer and its progression [48,49]. Future studies should confirm the downregulation of miR-5-7p and hence of TRIAP1 in the synergism of poziotinib and olmutinib. We conclude that combined therapies of poziotinib and olmutinib could be considerably beneficial for improving drug tolerance and overcoming the resistance of a single drug in lung cancer patients through extrinsic and intrinsic mechanisms regulated by hsa-miR-7-5p. However, it is important to emphasize that the effectiveness and safety of such treatments should be further evaluated in clinical trials and personalized patient care, considering individual variations in response and potential side effects.

The investigation into the combined administration of olmutinib and poziotinib for the treatment of non-small cell lung cancer (NSCLC) signifies a promising and progressive development within the realm of oncological research. It introduces a pathway toward more efficacious and personalized therapeutic strategies tailored to NSCLC patients, instilling optimism for enhanced clinical outcomes and a heightened quality of life. Prospective scientific inquiries, rigorous clinical trials, and healthcare initiatives will play a pivotal role in fully unraveling the therapeutic potential of this approach in the ongoing fight against lung cancer.

## 5. Conclusions

We conclude that poziotinib and olmutinib combination therapy shows synergistic action by activation and overexpression of the STK-11 gene which later associates with the p53-dependent extrinsic apoptotic pathway and by an increased Bax/bcl-2 ratio which activates the intrinsic apoptotic pathway. Both the pathways are activated and regulated by hsa-miR-7-5p. Cell lines employed in research may not entirely recapitulate the intricate and diverse nature of tumors encountered in patients. Consequently, the observations derived from A549 cells might not seamlessly extrapolate to clinical scenarios, thus constraining the broader applicability of these findings. Consequently, additional investigations utilizing animal models or clinical trials are imperative to substantiate the in vivo efficacy and safety of the combination therapy. This study thereby paves the way for future explorations and the prospective conduct of clinical trials to scrutinize the effectiveness of this combined therapeutic approach in individuals afflicted with non-small cell lung cancer (NSCLC).

## Figures and Tables

**Table 1:** Primer sequences of the genes used in gene expression.

Gene	Sequence of the Gene Expression Primers
Stk-11	F: 5′ GCCGGGACTGACGTGTAGA 3′
	R: 5′ CCCAAAAGGAAGGGAAAAACC 3′
Bcl-2	F: 5′ AATGGGCAGCCGTTAGGAAA 3′
	R: 5′ GCGCCCAATACGACCAAATC 3′
Bax	F: 5′ GGCCCTTTTGCTTCAGGGTT 3′
	R: 5′ GGAAAAAGACCTCTCGGGGG 3′
Gapdh	F: 5′ ACAGTCAGCCGCATCTTCTT 3′
	R: 5′ TTGATTTTGGAGGGATCTCG 3′

**Table 2:** Pharmacokinetic and Bioavailability characteristics of poziotinib and olmutinib.

Molecule	Olmutinib	Poziotinib
Canonical SMILES	C=CC(=O)Nc1cccc(c1)Oc1nc(Nc2ccc (cc2)N2CCN(CC2)C)nc2c1scc2	C=CC(=O)N1CCC(CC1)Oc1cc2c (ncnc2cc1OC)Nc1ccc(c(c1F)Cl)Cl
Formula	C26H26N6O2S	C23H21Cl2FN4O3
MW	486.59	491.34
H-bond acceptors	5	6
H-bond donors	2	1
TPSA	110.86	76.58
i Log P	4.07	4.13
X Log P3	4.75	5.36
W Log P	4.15	5.42
M Log P	3.03	3.76
Silicos-IT Log P	3.88	4.85
Consensus Log P	3.98	4.7
ESOL Log S	−5.77	−6.16
ESOL Class	Moderately soluble	Poorly soluble
Ali Log S	−6.81	−6.72
GI absorption	High	High
BBB permeant	No	No
Pgp substrate	Yes	No
CYP1A2 inhibitor	No	No
CYP2C19 inhibitor	Yes	Yes
CYP2C9 inhibitor	Yes	Yes
CYP2D6 inhibitor	Yes	Yes
CYP3A4 inhibitor	Yes	No
Log Kp (cm/s)	−5.9	−5.49
Lipinski violations	0	0
Ghose violations	2	2
Veber violations	0	0
Egan violations	0	0
Muegge violations	0	1
Bioavailability score	0.55	0.55
PAINS alerts	1	0
Brenk alerts	1	2
Synthetic accessibility	4.18	3.3
Lead-likeness violations	3	2

## Data Availability

Data will be available on request to the corresponding author.
